# Supplementary material for: Effects of Anemoside B4 on Plasma Metabolites in Cows with Clinical Mastitis
Source: Vet Sci. 2023 Jul 5;10(7):437. doi: 10.3390/vetsci10070437 (PMC10383794; doi:10.3390/vetsci10070437)
Supplement: Supplementary file 1 [file vetsci-10-00437-s001.zip › vetsci-2443914-supplementary.pdf]

Table S1. Composition of the diets for cows

Items	Lactation
Ingredient, % of DM	
Alfalfa hay	22.63
Corn silage	16.52
Steam-flaked corn	20.14
Brewer's grain	5.51
Soybean meal	4.86
Extruded-soybean	7.54
Wheat bran	4.88
Cottonseed	10.19
Fat powder	0.99
Premix[1]	0.60
Calcium hydrophosphate	1.49
Calcium powder	0.59
NaCO3	0.79
NaCl	0.74
Molasses	2.53
Total	100
Chemical composition, % of DM unless noted	

DM	50.36
CP	18.14
NDF	30.3
ADF	20.68
Ca	1.03
P	0.69
NEL, Mcal/kg of DM	1.71

[1]Formulated to contain (per kilogram of premix) 1100 KIU vitamin A, 360 KIU of vitamin D3, 6500 IU of vitamin E,2525 mg CU, 4200 mg Mn, 10025 mg Zn, 4150 mg Fe, 60 mg Co, 200 mg of biotin, and ≤ 10% water.

Table S2. Differential metabolites identified of CM-0 vs Healthy cows in the positive or negative mode

No.	Ions mode	Adduct	Metabolite	VIP	Fold change	−log (*p*)	*m/z*	RT(s)
1	ESI+	(M+H) +	L-Citrulline	4.79	0.61	4.05	176.10	767.28
2	ESI+	(M+H−2H_2O) +	Glycocholic acid	2.21	3.00	3.22	430.29	65.86
3	ESI+	(M+H) +	L-Kynurenine	1.81	0.52	3.04	209.09	516.58
4	ESI+	(M+H) +	PC(18:0/18:1)	3.76	1.86	2.24	788.61	76.47
5	ESI+	(M+H) +	N6-methyladenosine	1.06	2.75	1.97	282.12	241.95
6	ESI+	(M+H) +	L-Glutamine	3.62	0.68	1.97	147.08	730.46
7	ESI+	(M+H) +	Uridine	2.17	0.70	1.80	245.08	309.45
8	ESI+	(M−H+2Na) +	Thioetheramide-PC	1.90	0.82	1.63	780.55	75.93
9	ESI+	(M+CH3CN+Na) +	Gly-Arg	1.36	2.01	1.62	295.15	903.59
10	ESI+	(M+H) +	LysoPC(14:0)	4.04	0.68	1.59	468.31	391.32
11	ESI+	(M+H) +	Uracil	2.76	0.71	1.55	113.03	309.58

12	ESI+	(M+H) +	1-Aminocyclohexanecarboxylic acid	2.36	0.35	1.53	144.10	553.28
13	ESI+	(M+CH_3CN+H) +	Lys-Cys	1.13	2.24	1.50	291.15	922.06
14	ESI+	M+	Choline	1.75	1.39	1.50	104.11	521.61
15	ESI+	M+	trimethylammonium cation	1.76	1.21	1.47	146.12	737.54
16	ESI+	(M+H) +	2-Thiocytidine	1.75	0.85	1.46	260.07	337.57
17	ESI+	(M+H) +	Triethanolamine	2.26	0.26	1.46	150.11	267.54
18	ESI+	(M+H) +	D-Lactose	2.64	1.75	1.43	343.12	785.64
19	ESI+	(M+H) +	L-Histidine	2.11	0.73	1.36	156.08	849.21
20	ESI+	(M+H) +	L-Tryptophan	3.12	0.83	1.35	205.10	511.08
21	ESI+	(M+H) +	Phthalic acid Mono-2-ethylhexyl Ester	2.63	1.41	1.32	279.16	30.27
22	ESI−	(M−H) −	Norethindrone Acetate	1.74	0.49	2.78	339.20	67.99
23	ESI−	(M−H) −	Thymine	2.08	0.61	2.69	125.03	168.38
24	ESI−	(M−H) −	Phenol	2.07	0.34	2.32	93.03	58.09

25	ESI−	(M−H) −	Salicyluric acid	3.55	0.42	2.19	194.05	242.80
26	ESI−	(M−H) −	Salicylic acid	5.34	0.34	2.14	137.02	58.20
27	ESI−	(M−H) −	L-Valine	3.88	0.67	2.03	116.07	529.08
28	ESI−	(M−H) −	Oleic acid	4.49	2.37	1.96	281.25	81.15
29	ESI−	(M−H) −	Adynerin	1.30	0.63	1.96	515.30	66.01
30	ESI−	(M−H) −	L-Threonine	1.75	0.51	1.85	118.05	632.25
31	ESI−	(M−H) −	DL-lactate	4.01	1.64	1.80	89.02	393.88
32	ESI−	(M−H) −	PGF3a	1.03	1.49	1.78	351.22	166.98
33	ESI−	(M+Na−2H) −	Stavudine	1.11	2.63	1.75	245.05	533.66
34	ESI−	(M−H) −	Thymidine	2.71	0.68	1.69	241.08	168.31
35	ESI−	(M−H) −	Phenylacetylglycine	2.48	0.65	1.66	192.07	320.92
36	ESI−	(M−H) −	Pelargonic acid	2.33	1.37	1.66	157.12	74.86
37	ESI−	(M−H) −	20-HETE	1.32	2.63	1.44	319.23	44.04

38	ESI−	(M−H) −	Allantoin	4.76	0.91	1.43	157.04	314.03
39	ESI−	(M−H) −	L-Pyroglutamic acid	4.37	1.31	1.42	128.03	533.48
40	ESI−	(M−H) −	Dihydrothymine	1.20	0.69	1.34	127.05	671.11
41	ESI−	(M−H) −	Protocatechuie acid	4.18	0.50	1.31	153.02	38.91

Notes: ESI + was positive ion model, ESI− was negative ion model. Log(*p*) indicated the logarithmic function of p -value based on 10. −Log10 (0.05) = 1.3. m/z = mass-to-charge ratio; RT = retention time; VIP = variable importance in projection. The same below.

Table S3. Differential metabolites identified of CM-3 vs CM-0 cows in the positive or negative mode

No.	Ions mode	Adduct	Metabolite	VIP	Fold change	−Log (*p*)	*m*/*z*	RT(s)
1	ESI+	(M+H) +	PC (16:0/16:0)	1.59	0.54	3.00	734.56	309.22
2	ESI+	(M-H+2Na) +	Thioetheramide-PC	3.31	1.42	2.58	780.55	75.93
3	ESI+	(M+H) +	Creatinine	7.59	1.17	2.09	114.07	324.52
4	ESI+	(M+Na) +	LysoPC (18:1)	6.15	1.67	2.08	544.34	387.74
5	ESI+	(M+H) +	LysoPC (14:0)	4.44	1.68	1.91	468.31	391.32

6	ESI+	(M+Na) +	PC(18:0/18:1)	5.17	0.58	1.89	810.60	296.67
7	ESI+	(M+Na) +	LysoPC (18:0)	6.22	1.46	1.85	546.35	388.92
8	ESI+	$(M+CH_3CN+H)$ +	cis-9-Palmitoleic acid	4.91	2.13	1.58	296.26	55.65
9	ESI+	(M+H) +	N6-Methyl-L-lysine	1.81	0.62	1.41	161.13	1050.97
10	ESI+	(M+H) +	Phosphorylcholine	3.19	0.52	1.39	184.07	303.42
11	ESI+	$(M+H-H_2O)$ +	PC(18:1/18:1)	3.02	0.62	1.35	768.59	296.75
12	ESI+	M+	Glycerophosphocholine	2.53	0.77	1.32	258.11	757.52
13	ESI+	(M+H) +	Stearoylcarnitine	1.53	1.97	1.32	428.37	311.70
14	ESI−	(M−H) −	Isobutyric acid	1.24	0.25	2.02	87.04	226.63
15	ESI−	$(M-H_2O-H)$ −	15-keto-PGE1	2.48	2.02	1.78	333.21	219.61
16	ESI−	(M−H) −	Linoleic acid	1.12	0.63	1.68	279.23	168.91
17	ESI−	(M−H) −	Uridine	2.94	0.67	1.68	243.06	275.21
18	ESI−	(M−H) −	Salicyluric acid	2.04	0.20	1.58	194.05	242.80

19	ESI−	(M−H) −	Ginkgolic Acid	2.52	2.72	1.41	345.24	38.30
20	ESI−	(M−H) −	Eicosapentaenoic acid	2.74	0.66	1.35	301.22	64.52

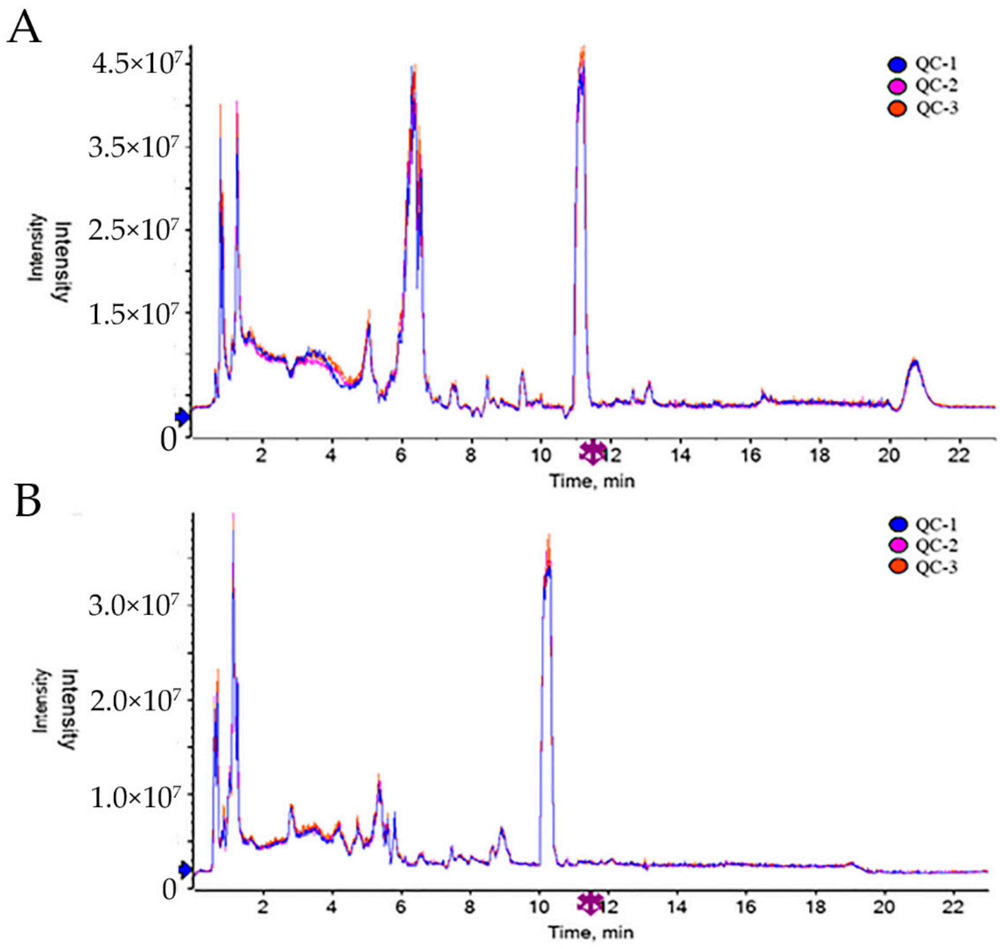

Figure S1. The total ion chromatograms (TIC) plot in (A) positive and (B) negative ion mode.

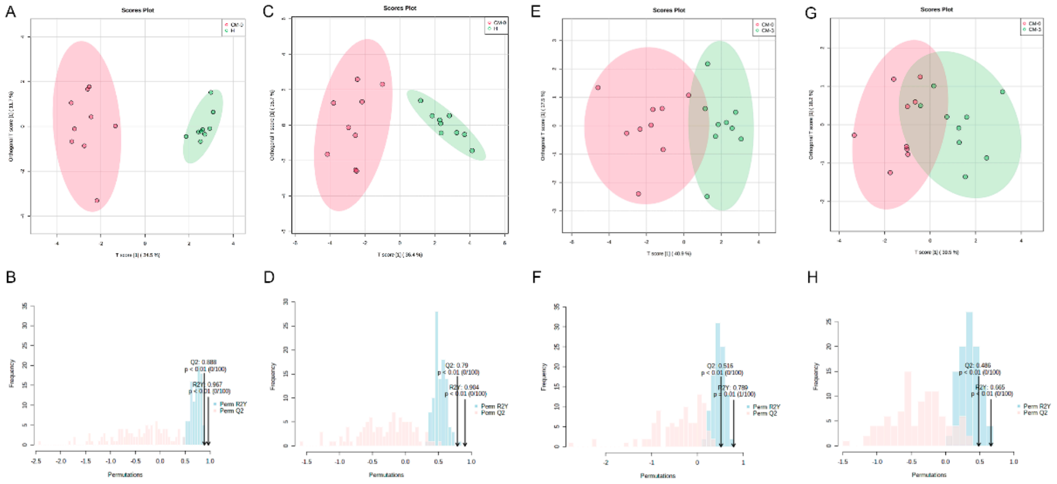

Figure S2. Orthogonal partial least squares discriminant analysis (OPLS-DA) of plasma metabolites and permutation test plots for the H and CM-0 group samples analyzed in the positive or negative ion mode (**A**, **B**) and (**C**, **D**). OPLS-DA of scores and permutation test plots for the CM-0 and CM-3 group samples analyzed in the positive or negative ion mode (**E**, **F**) and (**G**, **H**). The prediction parameters to evaluate the OPLS-DA model are R^2X, R^2Y, and Q^2. R^2X and R^2Y represent the interpretation rate of the model to the X and Y matrix, respectively, and Q^2 represents the predictive ability of the model. The R^2Y and Q^2values of values of > 0.4 indicated that the model was stable and reliable. H, healthy; CM, clinical mastitis; CM-0 is the day of diagnosis, CM-3 is the 3 consecutive days of anemoside B4 administration.
